# Questioning the application of risk of bias tools in appraising evidence from natural experimental studies: critical reflections on Benton et al., *IJBNPA* 2016

**DOI:** 10.1186/s12966-017-0500-4

**Published:** 2017-04-19

**Authors:** David K. Humphreys, Jenna Panter, David Ogilvie

**Affiliations:** 1 Department of Social Policy and Intervention, University of Oxford, 32 Wellington Square, Oxford, OX1 2ER UK; 2 Green Templeton College, University of Oxford, 43 Woodstock Road, Oxford, OX2 6HG UK; 3 MRC Epidemiology Unit and Centre for Diet and Activity Research (CEDAR), University of Cambridge, Box 285, Cambridge Biomedical Campus, Cambridge, CB2 0QQ UK

**Keywords:** Natural experiments, Built environment, Physical activity, Risk of bias

## Abstract

We recently read the article by Benton et al. which reviewed risk of bias in natural experimental studies investigating the impact of the built environment on physical activity (Benton et al., 2016; Int J Behav Nutr Phys Act 13:107). As a technical exercise in assessing risk of bias to understand study quality, we found the results of this study both interesting and potentially useful. However, it prompted a number of concerns with the use of risk of bias tools for assessing the quality of evidence from studies exploiting natural experiments. As we discuss in this commentary, the rigid application of such tools could have adverse effects on the uptake and use of natural experiments in population health research and practice.

## Background

We recently read an interesting article by Benton and colleagues published in *IJBNPA* [1]. The authors should be commended for adapting a new Cochrane Risk of Bias Tool—the ACROBAT-NRSI (or ROBINS-I)—for use in assessing the risk of bias when appraising natural experimental studies of the built environment [1, 2]. We agree with the authors’ calls to improve the rigour, execution and transparency of reporting in natural experimental studies [3–5]. Despite our admiration of this study, it prompts concerns about the application of rigid appraisal tools to studies exploiting natural experiments. As researchers with experience using natural experiments to generate policy relevant evidence, we are concerned that using tools like ACROBAT-NRSI without careful consideration of the practical difficulties in generating research evidence in certain areas of public health may serve to set the bar of methodological acceptability too high. This could lead to a widespread downgrading of evidence from natural experimental studies, further entrenching an existing ‘evaluative bias’ in favour of interventions that are easier to evaluate [6].

Research that exploits natural experiments is both opportunistic and pragmatic and investigators are often severely limited by practical circumstances. While the logic of natural experimental studies resembles the randomised controlled trial, we would argue that in practice they are quite different. Consequently, basic methodological features expected in prospectively designed trials (e.g. blinding or allocation concealment) might be impossible in studies exploiting natural experiments due to the researchers’ lack of control over the intervention. Therefore great care must be taken to consider the current state of the evidence as well as the contextual challenges that may limit the application of certain design features. In areas of population health that lack a strong research base, even severely limited observational designs exploiting natural experiments may provide an important advance in knowledge. Furthermore, assessing risk of bias in a single publication may be a misleading way of interrogating the plurality of the evidence base from well-designed natural experimental studies that combine several complementary study designs, datasets and analyses using approaches such as pattern matching or triangulation [7–9]. In such cases, the risk of bias for the entire evaluation may be low, even if the risk of bias in some individual analyses is high [10]. In the following sections we revisit the basic principles and purpose of natural experiments and consider the relevance of the ACROBAT-NRSI as a tool for appraising quality.

### The theory and practice of using natural experiments to generate public health evidence

Natural experiments are, by definition, events that occur outside the control of the researcher. They are not “conducted” or “designed”; on the contrary, they are discovered [11]. Natural experiments are events that naturally assign units (i.e. people, groups, places) to a particular treatment, intervention or exposure. They can include natural disasters, sudden economic shocks, changes to local or national policies, or changes to the built environment. Often it is not the event that researchers are interested in, but the circumstances it may (or may not) generate. For example, certain changes to the built environment may increase access to greenspace, reduce traffic congestion, separate cyclists from motor vehicles or make neighbourhoods more walkable. Researchers capitalise on the opportunistic changes in these factors to test theories about the causes of health behaviours or other phenomena by designing observational or quasi-experimental studies around the natural experiment. Where a change in exposure to a putative causal factor occurs, this should result in measurable changes in certain health behaviours (e.g. physical activity) when comparing populations that are exposed and unexposed to the changes.

There is a difference in opinion over what may legitimately be considered a natural experiment [12]. Some argue that the term ‘natural experiment’ should be confined to those events that naturally assign units to an intervention at random (or ‘as-if’ random) [11, 13]. However, others–including the Medical Research Council (MRC) guidance on natural experiments–take a much broader approach, stipulating only that studies capitalise on unplanned “variations in exposure” [14]. This latter approach has become more popular in population health research in recent years. It advocates a more flexible and inclusive approach to the study of events that result in natural variation in exposures, which might be evaluated using a wide range of methods.

In reality the practice of designing evaluative studies around natural experiments is fraught with challenges. If the natural assignment of units to an exposure occurs in ways that are non-random, studies may be subject to the same threats to validity as observational studies and may require complicated statistical controls for confounders [11]. In addition, many natural experiments will be evaluated retrospectively, severely limiting the opportunities to employ ‘gold standard’ design elements such as objectively-measured outcomes or the use of ‘well matched’ control groups—a point Benton et al. prioritise, but which is rarely straightforward [3]. The successful use of natural experiments often depends on co-operation with practitioners to identify opportunities, methodological creativity and a degree of good fortune. Given that the rigour of natural experimental studies can so often be determined by factors beyond the researchers’ control, one has to question the extent to which a risk of bias tool—such as the one proposed by Benton et al.—distinguishes between design weaknesses on the one hand, and practical obstacles in the context in which researchers are working on the other.

### The use of risk of bias tools for natural experimental studies

Benton et al. comment that “According to the principles of the ACROBAT-NRSI four fifths of included outcomes are ‘too problematic to provide any useful evidence on the effects of interventions’ and one fifth have ‘some important problems’” (p. 11). We do not question the technical assessment of bias diagnosed by the ACROBAT-NRSI tool, but we question the interpretation of results. Rather than contextualising these findings within the realistic possibilities in the selected field, it uses a benchmark that reflects common expectations across a much broader field of study (the so called ‘hierarchy of evidence’). This approach prompted Benton et al. to conclude that all studies are “too problematic” to draw evidence from, whereas an alternative interpretation might be that all studies contain risks of bias that are inherent in this complex and challenging area of research, yet we can be more confident about some studies than others. If the aim is to inform policy, a more pragmatic interpretation of the evidence (and its limitations) is required.

This observation is not limited to natural experiments. A similar study by Movsisyan and colleagues appraised a number of systematic reviews to examine the appropriateness of the Grading of Recommendations Assessment, Development and Evaluation (GRADE) tool for interpreting reviews of complex interventions [15, 16]. The authors found that systematic reviews of complex interventions were more frequently categorised as of “very low” quality than those of more “simple” (e.g. pharmacological) interventions. No single review of a “complex intervention” was deemed to be of “high” quality under the GRADE criteria, despite the presence of studies considered to be the “best possible evidence” in their respective fields [15, 17]. The authors concluded that GRADE, in its current format, may not be suited to the appraisal of such studies.

The approach proposed by Benton et al. has potentially serious implications. For example, it might severely downgrade innovative and influential studies like that of Merom et al., one of the first to evaluate the impact of changes to the built environment on physical activity [18]. This study incorporated a number of innovative features, including the creation of graded exposure comparison groups and longitudinal monitoring of bike traffic using counters. Not only did the authors measure both subjective and objective outcomes, they also tested mechanisms that might help to explain the outcomes and thereby support causal inference, such as awareness of the new cycle routes—a strength of the design not reflected in ACROBAT-NRSI. Although the rigour of this study was not comparable to that of a randomised controlled trial, it has undoubtedly been influential in prompting others to identify, evaluate, and develop similar natural experimental studies [4, 5, 19, 20].

One might question how a number of landmark natural experimental studies might fare under the adapted ACROBAT-NRSI proposed by Benton et al. Take for example John Snow’s study investigating the causes of death related to cholera in mid nineteenth century London [21]. The most compelling evidence initially provided by Snow comprised of little more than a cross tabulation of cholera death rates in homes serviced by two water companies, one of which had moved its source intake pipe upstream while the other continued to extract potentially contaminated water in central London. This sudden natural change in exposure enabled a test of Snow’s theory that cholera was a waterborne rather than an airborne disease [11, 22]. Viewed in the context of the evidence at that time, Snow’s is a classic example of a study opportunistically exploiting a natural experiment using a limited and pragmatic analytical approach, which generated crucial evidence of great theoretical and practical importance where few studies previously existed. Paradoxically, if these initial findings had been appraised using ACROBAT-NRSI, Snow’s analysis would likely have been classified as unreliable.

## Conclusion

We are not against the use of risk of bias tools in general. They can be used as a way of understanding the limitations of the evidence and justifying a call for methodological improvement—which, to their credit, is what Benton et al. have done in this paper. But much greater care is required in making sense of such appraisals for natural experimental studies. A unique feature of natural experimental studies is that key factors central to the design are outside the control of researchers. A more pragmatic approach would consider the wealth (or scarcity) of existing evidence and acknowledge the practical obstacles researchers may face. This would be aided by greater effort in documenting successful opportunities to exploit natural experiments, and the characteristics that may facilitate success (e.g. strong relationships with political or administrative bodies to identify opportunities, understand processes of assignment to interventions, or provide access to data, etc.). Consolidating information on these and other factors would help researchers recognise the most promising opportunities to exploit natural experiments and improve the rigour of the resulting studies.
